# Epidermal growth factor receptor kinase domain mutations are rare in salivary gland carcinomas

**DOI:** 10.1038/sj.bjc.6604875

**Published:** 2009-01-27

**Authors:** R Dahse, O Driemel, S Schwarz, J Dahse, K Kromeyer-Hauschild, A Berndt, H Kosmehl

**Affiliations:** 1 HELIOS Clinics Erfurt, Institute of Pathology, Nordhauser Street 74, Erfurt 99089, Germany; 2 Department of Oral and Maxillofacial Surgery, University of Regensburg, Franz-Josef-Strauß-Allee 11, Regensburg 93053, Germany; 3 Institute of Pathology, University of Erlangen, Krankenhausstr. 12, Erlangen 91054, Germany; 4 Faculty of Electronics and Information Technology, Ruhr University Bochum, Universitätsstr. 150, Bochum 44801, Germany; 5 Institute of Human Genetics and Anthropology, Friedrich Schiller University of Jena, Kollegiengasse 10, Jena 07740, Germany; 6 Institute of Pathology, Friedrich Schiller University of Jena, Ziegelmühlenweg 1, Jena 07740, Germany

**Keywords:** epidermal growth factor receptor, mutation screening, salivary gland carcinomas, targeted therapy

## Abstract

Activating mutations within the epidermal growth factor (EGFR) tyrosine kinase domain identify non-small cell lung cancer patients with improved clinical response to tyrosine kinase inhibitor therapy. Recently, we identified two EGFR mutations in a cohort of 25 salivary gland carcinomas (SGCs) by screening the tumour samples for the both most common hotspot mutations in exons 19 and 21 by allele-specific PCR. Here, we present a comprehensive sequencing analysis of the entire critical EGFR tyrosine kinase domain in 65 SGC of the main histopathological types. We found EGFR mutations in the tyrosine kinase domain to be a rare event in SGCs. No additional mutations other than the two known exon 19 deletions (c.2235_2249del15) in a mucoepidermoid carcinoma and an adenoid cystic carcinoma have been detected. Other putative predictive markers for EGFR-targeted therapy in SGCs might be relevant and should be investigated.

Malignant salivary gland neoplasms account for <0.5% of all malignancies and approximately 3–5% of all head-and-neck cancers (Milano *et al*, 2007). Salivary gland carcinomas (SGCs) represent a histopathologically heterogeneous group with different biological behaviour and clinical outcome. The main histopathologic types are (1) mucoepidermoid carcinoma that represents 29–34% of malignant tumours; (2) adenoid cystic carcinoma (ACC; 20%); (3) adenocarcinoma including acinic cell carcinoma; and (4) salivary duct carcinoma (Milano *et al*, 2007). Progress in understanding the cell biology of SGCs and detecting vulnerable molecular pathways may lead to the development of new targeted therapy options. Epidermal growth factor (EGFR; also known as ErbB1) is considered a possible key pathway for therapeutic molecules. Overexpression of EGFR has been shown in both salivary ACC and non-ACC (Agulnik *et al*, 2007; Ettl *et al*, 2008), and these findings have provided the motivation for trials with EGFR inhibitor-based therapies. Anti-EGFR agents include (1) monoclonal antibodies (cetuximab or erbitux, panitumumab), which block the binding of natural EGFR ligands, such as EGF or TGF-*α,* resulting in inhibition of downstream signal transduction pathways and (2) small molecule tyrosine kinase inhibitors (TKIs), which act by binding the ATP pocket within the kinase domain of the EGFR and impairing its catalytic activity (gefitinib, erlotinib and lapatinib). Cetuximab (erbitux) has been tested in two phase II studies in patients with recurrent and/or metastatic SGCs including mainly ACC, a cancer with generally poor outcome. In 50% of patients, clinical benefit (i.e., response or stable disease for 6 months) could be achieved (Milano *et al*, 2007; Locati *et al*, 2008). Gefitinib was associated with a 53% stable disease rate (10/19) in ACC, but had no effects in patients with salivary duct tumours and mucoepidermoid cancer (Glisson *et al*, 2005). Lapatinib, inhibiting ErbB1 and ErbB2 tyrosine kinases, was studied in a phase II trial and stabilized disease for greater than 6 months in 47% of ACC patients (Agulnik *et al*, 2007).

Earlier studies (Lynch *et al*, 2004; Paez *et al*, 2004; Eberhard *et al*, 2005; Riely *et al*, 2006a) had identified a subset of lung cancer patients with rapid and durable clinical responses to EGFR TKIs and found an underlying association between activating mutations in the EGFR tyrosine kinase domain and therapy response. Since then, four validated drug-sensitising mutations and three kinase domain mutations associated with drug resistance have been reported (Riely *et al*, 2006b). As with lung adenocarcinomas, mutations in the EGFR tyrosine kinase domain may be associated with clinical efficacy of EGFR inhibitors in the treatment of SGCs. Recently, we identified a mucoepidermoid carcinoma and an ACC with a TKI-sensitising EGFR exon 19 deletion mutation by screening EGFR exons 19 and 21 for the two most common hotspot mutations by an allele-specific PCR test (Dahse and Kosmehl, 2008).

The aim of this study was to sequence the entire critical EGFR tyrosine kinase domain and to determine how frequently drug-sensitising and drug-resistant EGFR mutations might occur in a large cohort of SGCs of the main histopathological types.

## Materials and methods

Surgically removed formalin-fixed tumour samples were obtained from 65 patients (35 males and 30 females with a median age at diagnosis of 55 years) treated with the histopathological diagnosis of an SGC according to the WHO classification (Barnes *et al*, 2005). All patients received surgery and in selected cases of postoperative radiation therapy. Epidermal growth factor receptor-targeted therapy was not applied. The study cohort consisted of ACC (*n*=25), mucoepidermoid carcinoma (*n*=10), myoepithelial carcinoma (*n*=8), acinic cell carcinoma (*n*=12) and adenocarcinoma ex pleomorphic adenoma (*n*=10).

Deoxyribonucleic acid was isolated from microdissected tumour areas using the QIAamp DNA Mini Kit (Qiagen GmbH, Hilden, Germany).

Epidermal growth factor exons 18, 19, 20 and 21 were amplified by nested PCR using primers described previously (Eberhard *et al*, 2005). Polymerase chain reaction products were purified with the QIAquick PCR Purification Kit (Qiagen GmbH). Genomic sequencing was performed using fluorescent dye-terminator chemistry and the primer M13F: 5′-TGTAAAACGACGGCCAGT-3′ (MWG Biotech, Martinsried, Germany). The reported mutations were scored as reproducible, having been identified in two independent PCR amplifications and by M13 sequencing in both directions (M13R: 5′-CAGGAAACAGCTATGACC-3′).

The numbering of the EGFR nucleotide and amino acid positions was according RefSeq sequences NM 005228.3 and NP 005219.2, respectively.

This study was conducted in accordance with the principles of the Declaration of Helsinki, as adopted by the 29th World Medical Assembly, Helsinki, Finland.

## Results

Sequencing analysis of exons 18–21 of the EGFR tyrosine kinase domain in 65 SGCs revealed two cases (3.1, 95% exact confidence limit: 0.5–1.2) with a heterozygous exon 19 deletion mutation. The two exon 19 E746_A750 deletions (both of them were variant c.2235_2249del15) were identified in a mucoepidermoid carcinoma of the left parotid gland grade 2 (54-year-old female patient, smoker), and in an ACC of solid type of the right parotid gland (69-year-old male patient, non-smoker). The female patient with the mucoepidermoid carcinoma was treated with surgery and postoperative radiation, the male patient with ACC by surgical tumour resection alone. Both patients had a favourable course of their cancer disease with no manifestation of recurrences (follow-up time 24 months).

In exon 20, an SNP c.2607 G>A (p.Q787Q) was detected in 29 out of 65 tumour samples (44.6%, 95% exact confidence limit: 32.5–57.4).

## Discussion

Somatic mutations in the EGFR kinase domain are found in a subset of lung adenocarcinomas and are associated with sensitivity to TKI (point mutations G719A/C in exon 18, L858R and L861Q in exon 21 and in-frame deletions at position 745 of exon 19). Three kinase domain mutations are associated with drug resistance: D761Y in exon 19, T790M in exon 20 and an exon 20 insertion (D770N771insNPG). The exon 19 deletions and L858R in exon 21 represent 85–90% of EGFR mutations in non-small cell lung cancer. In two recent trials in advanced non-small cell lung cancer, therapy with gefitinib administered in a genotype-directed fashion to patients harbouring EGFR mutations resulted in very favourable clinical outcomes with good tolerance (Sequist *et al*, 2008; Tamura *et al*, 2008).

Given the comparable embryonal tissue origin between lung adenocarcinoma and SGCs and the potential for genotype-directed trials of EGFR inhibitors, we undertook a comprehensive mutational analysis of EGFR in SGCs. We found EGFR mutations in the tyrosine kinase domain to be a rare event in SGCs of Caucasian origin. Whereas approximately 10–30% of lung carcinoma contain EGFR mutations (Mitsudomi and Yatabe, 2007), they seem to be seldom acquired in the cancers of other organs (EGFR mutation database:http://www.cityofhope.org/cmdl/egfr%5Fdb/).

Both of the mutations detected in our cohort were found in histopathological types (mucoepidermoid carcinoma and ACC), which also occur in the lung. In a recently published study, in two out of five (40%) of mucoepidermoid carcinoma of lung (which originate from minor salivary glands of the tracheobrochial tree), the L858R mutation was detected and all five patients well responded to TKI therapy independent of the mutation status (Han *et al*, 2008). In contrast, in another study with 24 cases of adenoid cystic and mucoepidermoid salivary gland-type lung carcinomas, EGFR protein expression was found in 91%, but no mutation was detected in exons 18–21 of the EGFR gene (Macarenco *et al*, 2008). In head-and-neck cancers, in which about 10% of cases showed responses to gefitinib, no mutations were noted in a US study, but 7% of tumours of Asian origin were found to harbour such mutations (Cohen *et al*, 2005; Lee *et al*, 2005).

In conclusion, drug-sensitising EGFR mutations are rather rare in SGCs and seem to be mainly found in a few mucoepidermoid and ACC. Taking into consideration the encouraging relative high number of responding SGC patients (particularly ACC patients with poor prognosis) who achieved stable disease after cetuximab treatment in the phase II trials and the apparently low incidence of drug-sensitising EGFR mutations, it would appear that also other biological factors than EGFR tyrosine kinase mutations might influence the therapy response. The underlying mechanisms of response to targeted therapies are complex and still not fully elucidated. Investigating those mechanisms and the mutation status of proteins in the cascade downstream of EGFR might identify further predictive markers for EGFR-targeted therapy in SGCs and would allow the selection of patients for a successful treatment.

## References

[bib1] Agulnik M, Cohen EW, Cohen RB, Chen EX, Vokes EE, Hotte SJ, Winquist E, Laurie S, Hayes DN, Dancey JE, Brown S, Pond GR, Lorimer I, Daneshmand M, Ho J, Tsao MS, Siu LL (2007) Phase II study of lapatinib in recurrent or metastatic epidermal growth factor receptor and/or erbB2 expressing adenoid cystic carcinoma and non adenoid cystic carcinoma malignant tumors of the salivary glands. J Clin Oncol 25: 3978–3984 17761983 10.1200/JCO.2007.11.8612

[bib2] Barnes L, Eveson W, Reichart P, Sidransky W (2005) Pathology and Genetics of Head and Neck Tumours. World Health Organization Classification of Tumours. IARC Press: Lyon, France, pp 210–242

[bib3] Cohen EE, Kane MA, List MA, Brockstein BE, Mehrotra B, Huo D, Mauer AM, Pierce C, Dekker A, Vokes EE (2005) Phase II trial of gefitinib 250 mg daily in patients with recurrent and/or metastatic squamous cell carcinoma of the head and neck. Clin Cancer Res 11: 8418–8424 16322304 10.1158/1078-0432.CCR-05-1247

[bib4] Dahse R, Kosmehl H (2008) Detection of drug-sensitizing EGFR exon 19 deletion mutations in salivary gland carcinoma. Br J Cancer 99: 90–92 18542074 10.1038/sj.bjc.6604430 PMC2453031

[bib5] Eberhard DA, Johnson BE, Amler LC, Goddard AD, Heldens SL, Herbst RS, Ince WL, Janne PA, Januario T, Johnson DH, Klein P, Miller VA, Ostland MA, Ramies DA, Sebisanovic D, Stinson JA, Zhang YR, Seshagiri S, Hillan KJ (2005) Mutations in the epidermal growth factor receptor and in KRAS are predictive and prognostic indicators in patients with non-small-cell lung cancer treated with chemotherapy alone and in combination with erlotinib. J Clin Oncol 23: 5900–5909 16043828 10.1200/JCO.2005.02.857

[bib6] Ettl T, Schwarz S, Kuhnel T, Stockmann P, Reichert TE, Driemel O (2008) [Prognostic value of immunohistochemistry in salivary gland cancer]. HNO 56: 231–238 18214402 10.1007/s00106-007-1666-x

[bib7] Glisson BS, Blumenschein G, Francisco M, Erasmus J, Zinner R, Kies M (2005) Phase II trial of gefitinib in patients with incurable salivary gland cancer. J Clin Oncol 23: 16S–5532

[bib8] Han SW, Kim HP, Jeon YK, Oh DY, Lee SH, Kim DW, Im SA, Chung DH, Heo DS, Bang YJ, Kim TY (2008) Mucoepidermoid carcinoma of lung: Potential target of EGFR-directed treatment. Lung Cancer 61(1): 30–34 18192072 10.1016/j.lungcan.2007.11.014

[bib9] Lee JW, Soung YH, Kim SY, Nam HK, Park WS, Nam SW, Kim MS, Sun DI, Lee YS, Jang JJ, Lee JY, Yoo NJ, Lee SH (2005) Somatic mutations of EGFR gene in squamous cell carcinoma of the head and neck. Clin Cancer Res 11: 2879–2882 15837736 10.1158/1078-0432.CCR-04-2029

[bib10] Locati LD, Bossi P, Perrone F, Potepan P, Crippa F, Mariani L, Casieri P, Orsenigo M, Losa M, Bergamini C, Liberatoscioli C, Quattrone P, Calderone RG, Rinaldi G, Pilotti S, Licitra L (2008) Cetuximab in recurrent and/or metastatic salivary gland carcinomas: A phase II study. Oral Oncol (e-pub ahead of print 17 September 2008) 10.1016/j.oraloncology.2008.07.010 18804410

[bib11] Lynch TJ, Bell DW, Sordella R, Gurubhagavatula S, Okimoto RA, Brannigan BW, Harris PL, Haserlat SM, Supko JG, Haluska FG, Louis DN, Christiani DC, Settleman J, Haber DA (2004) Activating mutations in the epidermal growth factor receptor underlying responsiveness of non-small-cell lung cancer to gefitinib. N Engl J Med 350: 2129–2139 15118073 10.1056/NEJMoa040938

[bib12] Macarenco RS, Uphoff TS, Gilmer HF, Jenkins RB, Thibodeau SN, Lewis JE, Molina JR, Yang P, Aubry MC (2008) Salivary gland-type lung carcinomas: an EGFR immunohistochemical, molecular genetic, and mutational analysis study. Mod Pathol 21: 1168–1175 18587327 10.1038/modpathol.2008.113 PMC2752817

[bib13] Milano A, Longo F, Basile M, Iaffaioli RV, Caponigro F (2007) Recent advances in the treatment of salivary gland cancers: emphasis on molecular targeted therapy. Oral Oncol 43: 729–734 17350323 10.1016/j.oraloncology.2006.12.012

[bib14] Mitsudomi T, Yatabe Y (2007) Mutations of the epidermal growth factor receptor gene and related genes as determinants of epidermal growth factor receptor tyrosine kinase inhibitors sensitivity in lung cancer. Cancer Sci 98: 1817–1824 17888036 10.1111/j.1349-7006.2007.00607.x PMC11159145

[bib15] Paez JG, Janne PA, Lee JC, Tracy S, Greulich H, Gabriel S, Herman P, Kaye FJ, Lindeman N, Boggon TJ, Naoki K, Sasaki H, Fujii Y, Eck MJ, Sellers WR, Johnson BE, Meyerson M (2004) EGFR mutations in lung cancer: correlation with clinical response to gefitinib therapy. Science 304: 1497–1500 15118125 10.1126/science.1099314

[bib16] Riely GJ, Pao W, Pham D, Li AR, Rizvi N, Venkatraman ES, Zakowski MF, Kris MG, Ladanyi M, Miller VA (2006a) Clinical course of patients with non-small cell lung cancer and epidermal growth factor receptor exon 19 and exon 21 mutations treated with gefitinib or erlotinib. Clin Cancer Res 12: 839–844 16467097 10.1158/1078-0432.CCR-05-1846

[bib17] Riely GJ, Politi KA, Miller VA, Pao W (2006b) Update on epidermal growth factor receptor mutations in non-small cell lung cancer. Clin Cancer Res 12: 7232–7241 17189394 10.1158/1078-0432.CCR-06-0658

[bib18] Sequist LV, Martins RG, Spigel D, Grunberg SM, Spira A, Janne PA, Joshi VA, McCollum D, Evans TL, Muzikansky A, Kuhlmann GL, Han M, Goldberg JS, Settleman J, Iafrate AJ, Engelman JA, Haber DA, Johnson BE, Lynch TJ (2008) First-line gefitinib in patients with advanced non-small-cell lung cancer harboring somatic EGFR mutations. J Clin Oncol 26: 2442–2449 18458038 10.1200/JCO.2007.14.8494

[bib19] Tamura K, Okamoto I, Kashii T, Negoro S, Hirashima T, Kudoh S, Ichinose Y, Ebi N, Shibata K, Nishimura T, Katakami N, Sawa T, Shimizu E, Fukuoka J, Satoh T, Fukuoka M (2008) Multicentre prospective phase II trial of gefitinib for advanced non-small cell lung cancer with epidermal growth factor receptor mutations: results of the West Japan Thoracic Oncology Group trial (WJTOG0403). Br J Cancer 98: 907–914 18283321 10.1038/sj.bjc.6604249 PMC2266849

